# Enhancing Adolescent Food Literacy Through Mediterranean Diet Principles: From Evidence to Practice

**DOI:** 10.3390/nu17081371

**Published:** 2025-04-17

**Authors:** Paula Silva

**Affiliations:** 1 Laboratory of Histology and Embryology, Department of Microscopy, School of Medicine and Biomedical Sciences (ICBAS), University of Porto (U.Porto), Rua Jorge Viterbo Ferreira 228, 4050-313 Porto, Portugal; psilva@icbas.up.pt; 2 iNOVA Media Lab, ICNOVA-NOVA Institute of Communication, NOVA School of Social Sciences and Humanities, Universidade NOVA de Lisboa, 1069-061 Lisbon, Portugal

**Keywords:** food literacy, adolescents, Mediterranean Diet, school-based interventions, Health Promotion

## Abstract

Adolescent obesity and inadequate dietary habits remain pressing public health concerns in Portugal, particularly among socioeconomically disadvantaged populations. Food literacy has emerged as a critical framework for promoting healthier eating behaviors; however, school-based interventions are rarely culturally grounded or theoretically structured. This narrative review synthesizes the experiential strategies described in the literature to improve adolescent food literacy in school settings. It provides a conceptual foundation for and supports the design rationale of FOODWISELab: The Mediterranean Diet Experience—a school-based intervention aligned with Mediterranean diet principles and specifically tailored to the Portuguese educational context. Findings from the literature—emphasizing the value of school gardens, cooking classes, digital tools, and curricular integration—guided the development of FOODWISELab: The Mediterranean Diet Experience, a comprehensive intervention designed for implementation in public secondary schools in Portugal. The proposed protocol bridges the gap between research and practice by offering a structured, context-sensitive model built around four core pedagogical domains: planning, selecting, preparing, and eating. It integrates multiple components, including hands-on learning, family and community involvement, and robust evaluation strategy. FOODWISELab addresses the well-documented gap in adolescent food literacy by delivering a feasible, culturally relevant, and replicable intervention. The anchored Mediterranean dietary model aims to promote adolescent health, sustainability, and cultural heritage in both the urban and rural educational contexts. By presenting a detailed and actionable protocol, this review enhances the practical value of food literacy research and offers strategic guidance for future educational and public health initiatives.

## 1. Introduction

Childhood and adolescent obesity are pressing public health concerns in Portugal as they affect a growing number of young individuals. Among adolescents aged 10–17 years, 8.7% were classified as obese and 23.6% as overweight [1]. Valente et al. (2024) [2] investigated the prevalence of overweight and obesity among Portuguese children aged 6 to 10, and found that 37% are affected, with a higher rate among boys. Boys also show a consistently higher body mass index (BMI) and body fat percentage across all age groups [2]. In addition to weight-related concerns, the cardiometabolic profile of Portuguese youth revealed additional risk factors. Coelho et al. (2024) [3] carried out a study within the Teen Without Risk project to assess the prevalence of obesity and related cardiometabolic risk factors among 156 adolescents aged 10–18 years from a school in Portugal. The study found that 13.5% of the participants were classified as obese and 17.3% as overweight, with boys showing a higher prevalence of excess weight. In addition, 38.8% of adolescents had elevated blood pressure, 62.2% had low high-density lipoprotein (HDL) cholesterol levels, and 34% had altered triglyceride levels. A significant association was observed between low-density lipoprotein (LDL) cholesterol and hypertension, and nearly 68% of the participants reported a family history of cerebrocardiovascular disease or diabetes [3]. These figures are closely linked to unhealthy dietary patterns marked by the insufficient consumption of fruits and vegetables and a high intake of sugary drinks, ultra-processed foods, and sweets [4]. The rising prevalence of obesity and related metabolic disorders in childhood and adolescence implies an urgent need for comprehensive and culturally responsive strategies that can address these health challenges at their roots.

The high rates of obesity among Portuguese adolescents point to underlying challenges in food literacy, highlighting the need for targeted interventions and educational strategies. As previously reviewed, among the various definitions presented in the literature, the most appropriate conceptualizes food literacy as a framework that empowers individuals, households, communities, and nations to maintain diet quality by adapting and enhancing dietary resilience over time [5,6]. This definition, proposed by Vidgen and Gallegos (2014), encompasses a set of interconnected knowledge, skills, and behaviors essential for planning, managing, selecting, preparing, and consuming food to meet dietary needs and determine intake [7]. In contrast, nutrition literacy pertains to the capacity to obtain, process, and recognize fundamental dietary information and services necessary for making informed food choices. It involves not only understanding nutritional concepts but also the ability to assess and apply nutrition-related information [5,6]. Food literacy offers a broader and more inclusive perspective than nutrition literacy, as the holistic nature of the definition of food literacy highlights the complexity of food-related behaviors and encourages comprehensive approaches to nutrition education and interventions [5]. Food literacy among Portuguese adolescents remains relatively limited, reflecting notable gaps in knowledge, attitudes, and behaviors that influence food choices. These findings suggest that many young people struggle to understand and apply the essential concepts that support healthy eating, highlighting the need for targeted educational strategies. While girls typically score higher than boys and older students outperform younger ones, overall literacy levels remain inadequate for fostering long-term health [8].

Portugal is renowned for its commitment to the Mediterranean Diet (MD), which is a key aspect of its cultural heritage. In December 2013, UNESCO recognized MD as an Intangible Cultural Heritage of Humanity, with Portugal being among the representative nations. This dietary pattern is rooted in the traditional eating habits of individuals in Crete and in other Mediterranean regions. MD is characterized by a high intake of vegetables, fruits, legumes, and whole grains, which provide essential nutrients, complex carbohydrates, and dietary fiber. Additionally, the diet is marked by limited total fat intake, with less than 30% of the total daily energy derived from fats, of which less than 10% comes from saturated fat. Monounsaturated fat is primarily sourced from olive oil, a fundamental dietary component. The emphasis of MD on fresh, minimally processed foods and their balanced macronutrient composition contributes to its reputation as one of the healthiest dietary patterns worldwide. Sustainability is also a key factor as it aligns with environmentally friendly agricultural practices and supports local food production. Given its well-documented health benefits and cultural importance, MD continues to be widely recommended as a model for healthy eating and a means of preventing chronic diseases [9]. MD is a healthy choice for individuals of all ages including adolescents. A systematic review and meta-analysis demonstrated that MD-based interventions can lead to small but significant reductions in BMI and obesity rates in children and adolescents. These effects are especially notable in those with excess weight and in Mediterranean countries, where cultural familiarity with the diet may enhance adherence and effectiveness [10]. These findings confirm that MD is an effective strategy for promoting healthy development and addressing the growing issue of childhood obesity. However, adherence to this dietary pattern remains low in many countries, including Portugal, where children and adolescents are increasingly moving away from core principles [11,12].

Farm to School (F2S) programs are multifaceted initiatives designed to connect students with local food systems while supporting healthier eating habits and enhancing educational experiences. At their core, F2S aims to bring locally or regionally produced food into school cafeterias, creating opportunities for students to engage with fresh, seasonal, and culturally relevant food. Although Farm to School (F2S) programs are not yet widespread in Portugal, they represent a pedagogical approach that strongly informs the FOODWISELab intervention model presented in this paper. These programs go beyond procurement; they are often accompanied by promotional efforts to encourage healthy eating as well as hands-on experiential learning activities such as school gardening, farm visits, and cooking demonstrations. The fundamental strength of the F2S framework lies in its integration into the academic curriculum, where agricultural and nutrition education is embedded within standards-based instruction, helping students to make meaningful connections between what they learn in the classroom and the food they consume daily. One of the defining features of the F2S framework is its flexibility. Rather than prescribing a one-size-fits-all model, it allows schools to tailor their activities to meet their specific goals, resources, and community contexts. This adaptability has contributed to their widespread appeal and implementation in diverse educational settings. Farm-to-school activities are typically organized into several key areas: sourcing and serving local foods, integrating farm-related content into the curriculum, offering experiential learning opportunities such as garden-based projects or field trips, promoting local foods through events and tastings, evaluating program outcomes such as food acceptance or waste, and training school food service staff to better implement these practices. Collectively, the F2S framework represents a dynamic approach to education and public health that not only nurtures student knowledge and behavior around food but also strengthens local economies and supports sustainable agriculture. Despite the growing body of work documenting their benefits, continued research is needed to assess their long-term impact on dietary habits, academic outcomes, and community resilience, particularly in underserved and urban school settings [13].

Enhancing food and nutrition literacy from an early age is fundamental to fostering healthy lifelong eating habits. Educational institutions, particularly schools, are positioned uniquely to support this process by offering structured and consistent learning opportunities. Adolescence is a critical period of development, defined by navigating challenging social circumstances and cementing identity as youth transition into emerging adulthood [14]. It is also a time of immense growth, second only to the first year of life, and, as such, nutrient requirements increase substantially [15]. These physiological and psychosocial transitions underscore the urgency of addressing food literacy during adolescence, because habits formed during this stage often track into adulthood and influence long-term health outcomes [16]. Although a range of school-based interventions, such as nutrition education programs and hands-on cooking classes, have proven effective in improving adolescents’ food-related knowledge, skills, and behaviors, a significant gap remains in the literature regarding school-to-farm interventions rooted in MD. Despite the well-documented health and sustainability benefits of this dietary pattern, particularly in Mediterranean countries such as Portugal, where it aligns with traditional eating practices, there is a lack of initiatives that integrate experiential learning in school farms with the MD principles. To address this gap, the following sections examine different types of school-based food literacy interventions implemented in various contexts. By analyzing their methodologies, outcomes, and contextual relevance, this narrative review aims to provide insights that inform the development of a culturally grounded school-to-farm intervention based on MD. Such a program would offer a sustainable and context-appropriate framework for promoting adolescent health and strengthening food and nutrition literacy in Portuguese educational settings.

## 2. School-Based Strategies to Promote Food Literacy in Adolescents

### 2.1. School Farm

School farms are distinct educational initiatives that combine experiential learning with agricultural practices, offering students the opportunity to engage directly with the food system through school-managed or school-affiliated agricultural sites. Unlike broader F2S programs, which encompass a range of activities to connect schools with local food producers, school farms represent a more specific approach that integrates food production into the educational environment [17]. These programs not only contribute to food literacy and local food security by providing fresh produce for school meals but also equip students with practical food-related skills. More than sites of food production, school farms function as dynamic learning environments that can be embedded across multiple academic disciplines and foster inclusivity among students from diverse and marginalized backgrounds [17,18].

The educational benefits of school farms extend beyond the acquisition of agricultural knowledge. Numerous studies have demonstrated that exposure to green spaces and hands-on activities in natural environments can enhance cognitive functioning, reduce stress, and improve emotional well-being. These outcomes translate into better academic performance, improved behavior, and stronger overall student engagement. Garden-based learning and green schoolyard initiatives have shown positive effects across various age groups, with particular gains observed in standardized science test scores. Particularly, at-risk students demonstrated lower failure rates, increased focus, and heightened motivation when engaged in these environments. The collaborative nature of farm-based education promotes the development of vital life skills, such as problem solving, teamwork, and communication, while also enhancing students’ ability to concentrate and regulate their emotions [19].

School farms also support broader goals related to social development, environmental stewardship and community engagement. Their interdisciplinary nature allows them to serve as outdoor laboratories, where concepts from environmental science, art, physics, and even industrial technology can be explored through experiential project-based learning [20]. Despite their potential, school farms are still underrepresented in the academic literature. A recent scoping review revealed that although school farms have a long history, once focused mainly on vocational agricultural training, modern discourse has shifted toward the themes of sustainability, healthy eating, and systems thinking. However, only 14 peer-reviewed empirical studies have been identified, and much of the existing body of work consists of editorials and gray literature [17]. This lack of scholarly attention highlights both the conceptual ambiguity surrounding the definition of a school farm and the urgent need for rigorous evaluation of its educational, environmental, and health impacts.

This gap in research is particularly evident in the absence of studies investigating how school farms might promote adherence to culturally and scientifically endorsed dietary patterns, such as the MD. Despite the widespread recognition of the health and environmental benefits of MD, no empirical research has examined its integration into school farms. This is especially notable given the growing popularity of school roof farms in urban areas, which could offer ideal conditions for cultivating Mediterranean staples such as vegetables, legumes, and herbs. These spaces hold great promise for combining experiential learning with nutrition education, while also supporting environmental awareness and appreciation for cultural food heritage. Strategically incorporating regionally appropriate diets into school farm programs could significantly strengthen their role in promoting healthy eating habits, particularly in urban settings where children may face both dietary risks and limited access to fresh food.

The future of school farms lies in their integration into broader, multifaceted educational strategies that address the systemic challenges in health, education, and sustainability. An isolated implementation is unlikely to have a lasting impact. Instead, school farms should be recognized as part of a more comprehensive pedagogical framework that includes curricular integration, community partnerships, and alignment with public health and sustainability goals. By embedding these initiatives within such frameworks, schools can maximize their educational value and meaningfully contribute to students’ well-being and community resilience.

### 2.2. School Gardens

School gardens are educational spaces located within school grounds that offer students hands-on opportunities to engage in gardening, nutrition and environmental education. Through planting, cultivating, and harvesting vegetables, students form tangible connections between food production and consumption, contributing to improved food literacy and a deeper understanding of sustainability and health [21]. These gardens serve not only as supplementary teaching tools but also as integral components of academic instruction, particularly in science and health education [22]. Research has consistently demonstrated that school gardens promote environmental awareness [23], enhance social and emotional learning [24], and improve children’s eating behaviors, including increased vegetable consumption [25]. Their implementation fosters scientific understanding across educational levels [26] and supports student well-being by creating spaces that connect learners to nature and one another.

School gardens offer multifaceted psychological benefits, particularly for adolescents, by supporting their mental well-being through their experiences of connection, personal growth, and health. Participatory research using photovoice methodology has shown that these gardens promote a sense of social belonging, inclusion, and identity, especially through collaborative gardening tasks. Food literacy is a key factor in this process. Students develop operational food literacy by planting and preparing food, cultural food literacy by engaging with diverse food traditions, and critical food literacy by reflecting on food systems and sustainability. These experiences build resilience, environmental consciousness, and a sense of autonomy. Activities such as working on the soil or harvesting food are often described as calming and empowering, while preparing meals from gardens fosters creativity and reinforces mastery. The combination of physical activity, sensory stimulation, and reflective engagement contributes to emotional regulation and stress relief [27].

The integration of school gardens into formal curricula has shown promising results in terms of shaping students’ food preferences. In one study, students who participated in weekly garden-based science lessons showed improved vegetable recognition, willingness to try new foods, and increased school-based vegetable intake. These effects were absent in the control group, which followed traditional instructions and experienced a decline in preference for vegetables. However, the influence of the intervention did not extend to eating behaviors at home, indicating the limitations of school-based programs that operate in isolation. These findings emphasize the importance of involving families and communities to sustain behavioral changes beyond the school setting. Furthermore, garden lessons addressed essential scientific concepts such as photosynthesis, soil health, and climate change, and were aligned with educational standards, demonstrating that garden-based instruction can effectively replace conventional lessons without sacrificing academic content. However, researchers have noted the need for more curriculum-aligned resources and interdisciplinary applications to maximize the educational value of garden-based learning [28].

Evidence from secondary schools in New Zealand offers additional insight into the potential health benefits of school gardens. A large-scale study found that students attending schools with gardens had lower BMIs and were less likely to be overweight, particularly socioeconomically disadvantaged students. This finding suggests that gardens may act as equity-promoting interventions. Interestingly, while associations with improved dietary choices, such as reduced fast food consumption, were identified, the study did not observe significant changes in fruit and vegetable intake or physical activity levels. These results challenge the assumption that gardens influence health primarily through dietary intake and suggest that the effects may occur via more complex psychosocial pathways, such as shaping food attitudes and fostering ownership of food production [29].

The addition of a school garden to nutrition education programs can further enhance behavioral outcomes. In Mexico, students who received both classroom-based food education and experiential garden sessions showed greater increases in fruit and vegetable consumption than those who received only theoretical instruction. This group also demonstrated improved dietary quality, including a lower overall caloric intake and healthier macronutrient profile. Students reported feeling more connected and motivated in the garden setting, describing it as a space in which they feel proud and engaged. The program’s curriculum, grounded in cognitive constructivism and aligned with national education standards, addressed themes such as healthy eating, food systems, and sustainability. The garden served as both an educational and motivational space, reinforcing theoretical content and encouraging real-world applications. Some students were inspired to start gardens at home, indicating a meaningful transfer of knowledge and behavior to family settings. However, even with these positive outcomes, the study acknowledged that students’ dietary intake remained below international recommendations, suggesting the need for longer or more intensive interventions and caution against overgeneralizing findings from privileged populations to more diverse contexts [30].

In resource-limited settings, school gardens have demonstrated potential as components of broader nutritional strategies. In a study conducted in Tanzania, school gardens were implemented as part of a multicomponent intervention that included school meals, nutrition education, and community engagement. In full-intervention schools, gardens produced supplemented school meals, directly contributing to students’ diets. In other cases, vegetables were sold to improve the school infrastructure or distributed to families during community events. Structured garden clubs allow adolescents to play an active role in cultivation, reinforce nutrition education, and promote practical agricultural skills. Although this study did not isolate the specific effects of school gardens, improvements in nutritional knowledge and dietary quality were observed among students and their families, suggesting an indirect value of the garden component. These findings reflect the potential of school gardens to contribute to behavioral change through experiential learning, even if short intervention periods may not be sufficient to affect physiological health markers such as growth or anemia status [31].

School gardens demonstrate considerable promise as tools to promote adolescent health, enhance academic learning, and support food literacy. However, their success depends on sustained implementation and integration into broader, multifaceted strategies, including community involvement, curricular alignment, and public health policy support. Isolated interventions may yield modest or short-lived results, while comprehensive approaches can amplify their impact by addressing the social, cultural, and environmental factors that shape behavior. In this context, implementing a school garden based on MD principles offers an especially powerful opportunity. The MD pattern, centered on vegetables, legumes, fruits, whole grains, herbs, and olive oil, naturally complements crops grown in many school gardens, particularly in the Mediterranean or similar climates. Embedding this model into garden-based learning provides a culturally relevant and evidence-based framework for teaching nutrition, sustainability and food heritage. Through hands-on activities such as growing seasonal produce and preparing traditional meals, students can translate theoretical dietary recommendations into meaningful daily habits. This approach not only reinforces healthy eating behaviors and environmental stewardship but also fosters a deeper connection to local agricultural traditions and community food cultures. By integrating school gardens with MD, educational institutions can create coherent and context-sensitive nutrition education programs that support long-term dietary changes and contribute to the prevention of chronic diseases in children and their families. Thoughtfully embedded within broader educational ecosystems, these gardens can become powerful agents of transformative change for individuals and communities.

### 2.3. Cooking Classes

Cooking classes play a crucial role in improving food literacy and promoting healthier eating habits in adolescents. These educational experiences foster the development of practical cooking skills and self-efficacy, which are both closely associated with healthier dietary behaviors [32]. Adolescents who engage in cooking programs often demonstrate increased confidence in food preparation, a deeper understanding of nutrition, and a greater likelihood of making health-conscious food choices [33]. Research shows that participation in these classes is linked to higher consumption of fruits and vegetables among both boys and girls, although differences in baseline competencies and learning trajectories between genders suggest that instruction should be inclusive and adaptable to diverse needs [34]. Moreover, cooking classes serve as a vital platform for addressing the widespread lack of basic food literacy skills among teenagers, particularly when modern consumers often face low levels of nutritional knowledge and limited family support for healthy food practices [35]. These interventions not only offer immediate health benefits but can also help adolescents develop a more meaningful and long-term relationship with the food system. By embedding cooking into the school curriculum, educators can create a comprehensive food education experience that prepares young people to navigate and improve their dietary patterns in a sustainable and informed manner.

Several effective cooking class interventions have demonstrated promising results in enhancing adolescents’ food literacy and related behaviors (Table 1). The most common types of interventions implemented in studies aimed to improve food literacy among adolescents involved in practical cooking and food preparation skills. Most programs focus primarily on teaching hands-on cooking techniques and meal preparation [36,37,38]. While many of these programs are implemented in school contexts, such as after-school food and cooking clubs [36,37] and garden-integrated cooking programs [39,40], some are implemented in alternative educational settings. For example, one intervention was delivered as part of the Classic Upward Bound summer program at the University of Maine, where high school students participated in cooking sessions while residing on a university campus during a two-week period in July 2024 [38]. Community-based cooking classes and culinary camps are frequently used [37,41,42]. Many cooking clubs and camps feature culinary themes inspired by various cultural traditions, leveraging this diversity to heighten engagement and sustain interest among participants. Although the specific goals of each program vary, their objectives have common threads. Most interventions seek to improve practical cooking abilities, aiming to enhance participants’ confidence and competence in preparing meals [36,37,38]. Nutrition education is also a core element, often delivered in tandem with cooking activities, to increase the participants’ understanding of healthy food choices [38,39,40,41,42]. Several programs have aimed to boost self-efficacy and cultivate beliefs in adolescents that they can successfully prepare meals [36,37,41]. Some studies have also aimed to promote healthier dietary behaviors, particularly with respect to increasing fruit and vegetable consumption. In broader terms, many interventions have been designed to foster food literacy, encompassing knowledge and skills related to food selection, preparation, and the understanding of nutrition-related languages and concepts [38,39,40,42]. While many interventions have demonstrated favorable outcomes aligned with these goals, substantial changes in overall dietary quality or home cooking frequency have not been consistently observed across studies. The outcomes of these interventions were assessed using various methods, most often through pre- and post-intervention evaluation. Questionnaires are the most widely used to measure changes in attitudes toward cooking, self-efficacy, and nutrition knowledge [36,37,38,39,41]. Changes in dietary behavior were also assessed using techniques such as 24 h dietary recalls, food behavior surveys, and Likert-type scales to evaluate cooking techniques [40,41]. In addition to quantitative assessments, some programs include qualitative methods such as interviews or focus groups with participants, parents, and facilitators to gain more nuanced insights into participants’ experiences [36,38,41,42]. In terms of the impact, most programs reported partial success in meeting their goals. The outcomes included increased nutrition and food knowledge, greater self-efficacy, enhanced perceived cooking ability, and the adoption of new cooking techniques [36,37,41]. McAleese and Rankin (2007) documented increased daily servings of fruits and vegetables as well as participants preparing recipes learned during the program at home [40]. However, improvements in overall diet quality were limited, and no study has reported significant changes in the frequency of home cooking. Heterogeneity in the intervention structure further complicates the assessment of their broader impacts.

Several recommendations have been proposed to enhance the effectiveness of future food literacy programs for adolescents. Programs with a behavioral rather than purely educational focus tend to yield stronger outcomes in dietary behavior. Tailoring content and delivery to the developmental stages of adolescent participants is advised, with more complex content appropriate for older adolescents and foundational skills emphasized for younger ones [37,39]. The use of technology, such as internet platforms, computer applications, and social media, has been recommended to better engage this age group. Gender-specific interventions may also be beneficial given the differences in food choices and cooking self-efficacy between boys and girls. Cultural sensitivity and relevance are crucial, as dietary behaviors and attitudes toward food vary across ethnic groups. A more comprehensive approach integrating culinary messages, nutritional education, and school meal planning is recommended. Practical hands-on components such as food handling, tasting, and take-home meal samples should be maintained or expanded. Collaboration among chefs, home economists, and dietitians can enhance program design and delivery. Developing age-appropriate and behavior-specific evaluation tools is essential to accurately measure program outcomes and further refine food literacy interventions for adolescents [43]. Finally, there is a growing consensus that cooking education should not be limited to skill acquisition alone but should be integrated with broader food literacy content, including knowledge about food systems, cultural diversity, and sustainability, to prepare adolescents to make informed and responsible food choices [44].

Da Rocha Leal et al. (2011) [45] explored the relationship between the cooking habits and skills of Portuguese adolescents and their adherence to the MD. The findings revealed that nearly 10% of adolescents reported that they did not know how to cook, and the majority had never cooked vegetables, fish, or soups. Girls were more frequently involved in cooking activities than boys were, and families emerged as the primary source of culinary learning. Regarding dietary patterns, the KIDMED index indicated that 7.2% of the participants had poor adherence to MD, 50.8% showed average adherence, and 42.1% demonstrated good adherence. Importantly, adolescents with higher KIDMED scores were also more likely to report better cooking knowledge, more frequent engagement in cooking, greater enjoyment of culinary activities, and a stronger motivation to improve their skills. These findings suggest that acquiring and practicing cooking skills may contribute to healthier food choices and better adherence to MD. The authors recommended a greater emphasis on culinary education at home and in schools, highlighting the role of these environments in shaping long-term dietary habits [45].

Recent studies among university populations have provided further evidence that cooking and food skills are critical components in promoting adherence to MD. Among Turkish university students, cooking and food skills and access to kitchen facilities were positively associated with better dietary adherence. Women and students enrolled in health-related faculties reported higher levels of cooking and food skills, and greater adherence to Mediterranean dietary principles. Notably, food skills, particularly those involving label reading, consumer awareness, and resourcefulness, showed stronger correlations with diet quality than cooking skills alone. These results reinforce the idea that food literacy extends beyond the ability to cook and includes navigating the food environment and making informed choices. Access to kitchen facilities plays a particularly important role in enabling students to translate their knowledge and skills into practice. Students with such access had higher MD Adherence Screener scores, cooked more frequently, and consumed more home-prepared meals. The availability of kitchen facilities is one of the most significant variables explaining variance in adherence, supporting the view that structural conditions can either enable or hinder the application of food literacy in everyday life. These findings highlight the potential benefits of integrating skill development and infrastructural support into university-based health promotion strategies [46].

While these studies provide valuable insights into older adolescents and young adults, there remains a critical gap in the literature regarding school-based culinary interventions targeting younger adolescents. Although numerous educational programs have promoted healthy eating in school settings, few have placed cooking at the center of the learning experience, and even fewer have done so with the explicit goal of fostering understanding and practical application of Mediterranean dietary principles. However, evidence from other contexts supports the potential of culinary interventions to improve dietary behavior. For example, the Community Culinary Coaching Program implemented in a rural Israeli kibbutz demonstrated that hands-on culinary education, community engagement, and participatory goal setting can shift food purchasing patterns toward the increased use of vegetables, legumes, whole grains, and fish. Although not focused on adolescents or schools, the program involved children and adolescents in various communal food settings and achieved measurable changes in adherence to MD principles [47]. Similarly, a randomized controlled trial evaluating a family-centered culinary medicine education program found that hands-on kitchen-based instruction significantly improved adherence to MD compared with traditional nutrition counseling. Participating families were nearly three times more likely to adopt Mediterranean dietary patterns and reported increased consumption of fruits, vegetables, and whole grains, along with reduced food costs, highlighting that this dietary approach can be both practical and affordable, even for low-income populations [48].

These examples, though not school-based, offer compelling evidence that integrating experiential cooking education with nutrition messaging can lead to meaningful behavioral changes. They also underscored the feasibility and effectiveness of these programs across diverse populations and settings. Given the developmental importance of adolescence in establishing lifelong dietary habits, the school environment is a promising platform to implement similar interventions. Future research should therefore explore how culinary education rooted in MD can be adapted and introduced in school settings, with the aim of strengthening food literacy, fostering healthier behaviors, and promoting long-term well-being.

### 2.4. Curriculum

In contrast to the experiential components such as school gardens and cooking activities discussed earlier, this section focuses on formal curriculum-based approaches that aim to integrate food literacy into secondary education through structured, subject-aligned programs. Adolescence represents a critical window for shaping lifelong dietary behaviors, marked by substantial physical, cognitive, and social development. It is second only to infancy in terms of growth and nutritional requirements, and it is during this stage that young people begin to navigate complex food environments and exercise, increasing autonomy over food-related decisions [49]. However, despite the pressing need to foster health-promoting behaviors during this period, poor dietary habits remain prevalent, contributing to rising rates of obesity, diet-related diseases, and broader social, economic, and environmental concerns [50]. Schools are uniquely positioned to address this challenge by equipping adolescents with essential life skills through food literacy. However, the integration of food literacy and its necessary complement into secondary education remains limited in scope and depth. Current educational offerings are often confined to Home Economics or Health and Physical Education (HPE), resulting in fragmented and inconsistent food education delivery. Teachers in Queensland, Australia, for example, reported significant barriers within the design and technology curriculum, citing vague achievement standards, a lack of practical skill descriptors, and the misplacement of nutritional theory in HPE instead of Home Economics. A majority of Home Economics teachers (80%) advocate curriculum reform, emphasizing the need to include the term “food literacy” in official documentation, relocate nutritional content to appropriate subject areas, and make achievement standards clearer and more context-specific [49]. Despite these barriers, there are promising opportunities to expand food literacy education using a cross-curricular approach. Mapping the Australian secondary curriculum revealed that nearly 50% of the subjects have the potential to incorporate food literacy and numeracy. Disciplines such as Mathematics, Science, Environmental Studies, and Technologies, especially in the food context, are particularly well suited to this integration. A cross-disciplinary model would allow food-related concepts to be taught through various lenses, supporting a more holistic understanding of health, sustainability, and responsible consumption [50]. Practical and experiential learning are crucial for the success of such initiatives. Studies have shown that separating food theory from hands-on experience can lead to poorly contextualized or even counterproductive messages. Therefore, effective programs must include activities, such as cooking, gardening, label reading, and food system analysis. Digital tools also offer valuable support for students and teachers, facilitating the dissemination of research-informed content in ways that resonate with students’ lived experiences [50]. One example of a promising intervention is the Teens CAN: Comprehensive Food Literacy in Cooking, Agriculture, and Nutrition program in the United States. Developed for high school-aged students, Teens CAN offers a developmentally appropriate curriculum grounded in Social Cognitive Theory, Constructivism, and Backward Design. Its modular structure combines nutrition education, cooking, and agricultural literacy, delivered through experiential and application-based lessons. Nutrition modules emphasize adolescent-relevant nutrients and balance based on MyPlate guidelines; cooking sessions develop essential skills such as food safety and budgeting; and agricultural lessons connect students to the origins of food and the importance of minimally processed choices. Piloted in two low-income communities, the curriculum demonstrated strong potential to improve food literacy and health behaviors among adolescents [51]. However, even well-designed programs, such as Teens CAN, have limitations. A curriculum for the treatment of cultural diversity remains underdeveloped. Although translated into Spanish and sufficiently flexible to accommodate vegetarian diets and some family preferences, the lack of intentional integration of diverse culinary traditions may reduce its relevance in multicultural contexts. This highlights the need for future iterations to better reflect the cultural identities and foodways of underserved communities, an opportunity to make food literacy more inclusive and impactful [51]. Implementing such programs on this scale presents logistical challenges. Time constraints, curriculum overload, inconsistent food policies, and limited teacher training are significant barriers. Many educators lack the confidence or qualifications to deliver food and nutrition education effectively, especially outside of traditional subject boundaries. To overcome this, food education should be embedded in teacher training programs, accompanied by sustained professional development [50]. Equally important is the allocation of adequate resources and the establishment of supportive food environments within schools. Monitoring systems, particularly in settings such as school canteens, can reinforce adherence to healthy food policies and strengthen the consistency of educational messages [50]. Therefore, family engagement is essential. Adolescents’ food behaviors are strongly influenced by their home environment, and negative parental attitudes can undermine school-based efforts. Programs that foster meaningful collaboration between schools and families can help align food literacy messages and reinforce them beyond the classroom [50]. Ultimately, embedding food literacy and numeracy across the secondary school curriculum is a feasible and necessary step toward improving adolescent dietary behaviors. A well-supported, flexible, and culturally responsive framework would not only equip young people with the knowledge and skills needed for healthy living but also prepare them to navigate the broader food system with confidence and critical insight. Such a model requires educational reform at all levels, intersectoral collaboration, and a long-term commitment to building capacity among educators and communities. By prioritizing food education in adolescence, we can lay the foundation for a healthier, more equitable, and more sustainable future.

## 3. FOODWISELab: The Mediterranean Diet Experience

As discussed in the previous sections, Portugal faces a significant public health challenge, marked by high levels of adolescent obesity and low levels of food literacy. Adolescence represents a critical developmental window for shaping lifelong dietary behaviors, offering a timely opportunity to promote informed and health-conscious food choices, and reduce the risk of chronic diseases. Based on these findings from the literature reviewed, we developed FOODWISELab: The Mediterranean Diet Experience, a food literacy initiative rooted in the MD principles. The project is scheduled to be implemented in northern Portugal during the academic year, engaging adolescents from different types of school settings in urban centers. We hope that this pilot will serve as a foundation for broader adoption, with the potential for replication in other regions of Portugal and in Mediterranean and non-Mediterranean countries.

### 3.1. Study Setting

FOODWISELab: The Mediterranean Diet Experience will take place in public secondary schools located in the northern region of Portugal, involving different urban center types such as Porto and inland cities such as Vila Real. This region offers a rich sociocultural backdrop for exploring Mediterranean food literacy and for combining diverse urban lifestyles. Urban areas are facing increasing challenges related to ultra-processed food consumption and disconnection from traditional eating habits [52]. By including both settings, the project aims to promote equitable access to MD education and sustainable food practices among adolescents aged 12–18 years, addressing different social, cultural, and environmental challenges that influence dietary behaviors in Portugal.

### 3.2. Study Design

FOODWISELab: The Mediterranean Diet Experience is designed as a multi-arm, school-based randomized controlled trial (RCT) to be implemented over one academic year. The intervention will involve six public secondary schools, selected through a stratified sampling approach, to ensure representation across different socioeconomic backgrounds, urban settings, and school sizes. Within each stratum, schools will be matched in pairs based on key characteristics and randomly assigned to one of three groups: full, partial, or control. The full intervention group will receive a comprehensive package, including the creation or revitalization of school gardens; hands-on cooking workshops aligned with MD principles; digital food literacy activities; and community-based workshops involving students, teachers, and families. The partial intervention group will receive the same components, excluding the cooking workshops. The control group will maintain standard educational practices without any additional FOODWISELab activities. This design reduces selection bias, accounts for contextual diversity, and allows for a robust evaluation of both the individual and the combined effects of the intervention. Specifically, it enables an assessment of the added value of hands-on cooking experiences in broader food literacy education. The pilot will be implemented throughout the academic year, laying the groundwork for further evaluation, adaptation, and potential scaling up.

### 3.3. Selection of Schools

Eligible public secondary schools will be identified through a stratified sampling strategy, ensuring balanced representation across the following key variables: (1) urban location, (2) school size (based on total student enrollment), and (3) socioeconomic profile (based on regional indicators). Schools within each stratum must also meet the following inclusion criteria: (a) no existing school gardens or structured food literacy programs, (b) access to outdoor spaces suitable for gardening, and (c) reliable year-round water availability. Following the stratified selection process, six schools will be matched in pairs based on similarities within the strata. Each matched pair will then be randomly assigned to one of the three arms: full intervention, partial intervention, or control. This process will ensure that the demographic and contextual factors that may influence outcomes are accounted for, supporting both the internal and external relevance of the findings. The design also facilitates meaningful comparisons across diverse school environments in northern Portugal, strengthening the evidence base for future policy and practice in food literacy education [53].

### 3.4. Selection of Participants

The Mediterranean Diet Experience will include adolescents enrolled in public secondary schools. The inclusion criteria are as follows: (1) enrollment in the equivalent of grades 7 to 10 (typically corresponding to ages 12 to 16 in Portugal), (2) written informed consent provided by a parent or legal guardian, and (3) informed consent provided by an adolescent. Participants must also be able to communicate effectively in Portuguese, as all intervention components will be delivered in the local language.

The intervention will be implemented at the classroom level and will be designed for all adolescents, regardless of their nutritional status. As such, the program includes students with diverse profiles, including those who are overweight, obese, or of a normal weight, and aims to promote food literacy as a universal competence. Although the FOODWISELab intervention adopts a universal approach, targeting all classroom groups, it acknowledges that food literacy levels, motivations, and learning needs can vary significantly among adolescents. Therefore, the program incorporates pedagogical flexibility through differentiated activities, modular content, and active learning strategies. This allows facilitators to tailor engagement to diverse student profiles, including those with overweight or obesity, without stigmatization. Drawing from diagnostic assessments and classroom dynamics, educators can adjust the level of complexity or emphasis on specific components (e.g., nutrition basics, food system analysis, or cooking skills), ensuring that the intervention remains relevant and inclusive. By promoting a responsive learning environment, FOODWISELab aims to maximize its impact across heterogeneous adolescent populations.

For each participating student, one parent or guardian will be invited to join the community workshop component of the intervention, foster intergenerational learning, and extend the impact of the program into the home environment.

### 3.5. Intervention Framework

**Figure 1:**
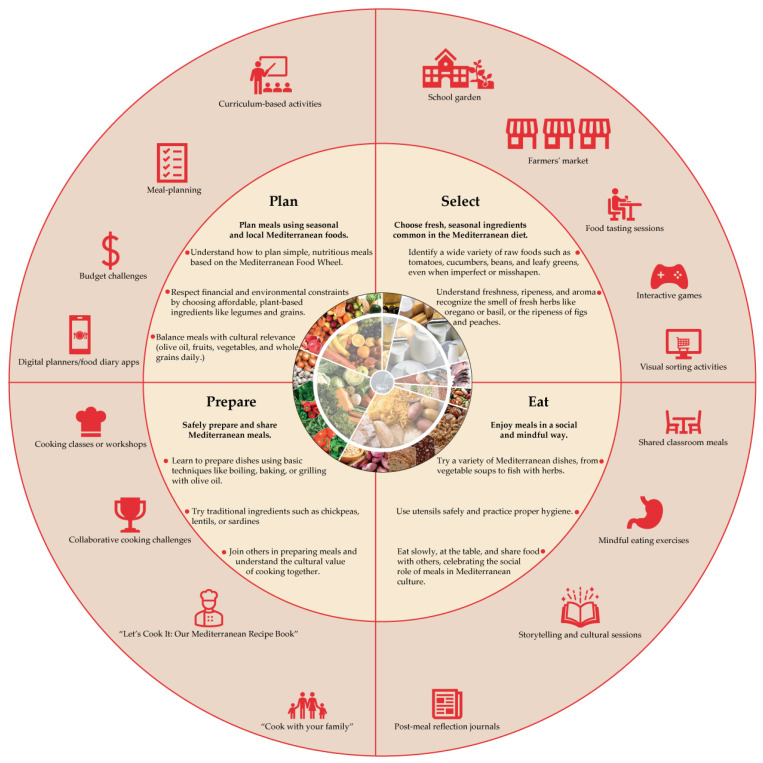
FOODWISELab pedagogical framework: Plan, Select, Prepare, and Eat. This figure illustrates the four core pedagogical domains of the FOODWISELab: The Mediterranean Diet Experience intervention—Plan, Select, Prepare, and Eat—each representing a key dimension of adolescent food literacy education. Around each domain, examples of related learning activities are provided, including school gardens, meal planning exercises, cooking workshops, farmers’ market simulations, digital tools, and community-based initiatives. These activities are designed to foster cognitive, behavioral, and emotional engagement with the principles of the Mediterranean Diet.

FOODWISELab: The Mediterranean Diet Experience intervention is grounded in a comprehensive food literacy framework inspired by the MD and organized into four interconnected domains: Plan, Select, Prepare, and Eat [43]. These domains serve as the pedagogical backbone of the program and are operationalized through a series of digital and experiential educational tools designed to engage adolescents both inside and outside the classroom. Educational resources and activities were selected based on the literature reviewed in this article, with an additional alignment with the pedagogical and technological requirements defined in the FOODWISELab platform development plan. The intervention adopts a multimodal and interactive approach, combining school-based learning environments with digital tools and community engagement strategies to promote meaningful, sustained learning [54] (Figure 1). Each of the four domains corresponds to a set of learning outcomes and the associated educational tools.

- Plan: Students learn to plan meals using seasonal and local ingredients aligned with the Mediterranean Food Wheel. Activities include meal budgeting simulations, interactive games, digital food diaries, and curriculum-linked sessions on balanced eating and environmental sustainability.
- Select: Adolescents are guided to recognize a variety of foods, understand seasonality, identify plant-based ingredients, and reflect on their food choices. Educational tools include food tasting sessions, farmers’ market simulations, visual sorting exercises, and food labeling challenges designed to strengthen informed food selection skills.
- Prepare: Hands-on cooking workshops, recipe co-creation activities, and team-based culinary challenges are used to foster basic food preparation skills, food safety awareness, and cultural connections through traditional Mediterranean dishes. This component promotes collaboration, autonomy, and confidence in the kitchen.
- Eat: This domain emphasizes mindfulness and social eating practices. Activities include shared classroom meals, storytelling linked to culinary heritage, post-meal reflection journals, and family-based initiatives such as “Cook with your family” or collaborative contributions to “Let’s Cook! Our Mediterranean Recipe Book.”

All activities are designed to be age-appropriate for adolescents aged 12 to 18 years and adaptable to various school contexts. The integration of these tools will occur primarily through extracurricular sessions led by trained educators and supported by the FOODWISELab Digital Platform. The platform will also host supplementary resources, including eBooks, educational games, and digital storytelling tools, to ensure continuity of learning beyond the classroom and to reinforce the core values of MD: simplicity, seasonality, conviviality, and sustainability. Importantly, the intervention actively encourages family engagement, recognizing that the support and involvement of parents and caregivers are essential to reinforce healthy eating behaviors and ensure the long-term success of the program. To this end, specific workshops and tailored digital educational materials will be developed for parents and caregivers, fostering their active participation and extending the impact of the intervention to the home environment. This integrative structure ensures that the intervention addresses not only the cognitive aspects of food literacy, but also the social, emotional, and behavioral dimensions, offering a scalable and adaptable model that may inform broader food education policies and practices.

### 3.6. Evaluation Strategy

The Mediterranean Diet Experience intervention will be evaluated using an enhanced mixed-methods longitudinal design that combines quantitative and qualitative data collection at multiple time points to capture both short- and long-term impacts. Baseline assessments will be conducted before implementation to establish the initial levels of food literacy, dietary habits, and relevant socioeconomic factors. These data will serve as a reference for evaluating changes attributable to the intervention. Quantitative data will be collected at baseline, end-line (after one academic year), and at follow-up intervals of 6 months, 1 year, and 2 years post-intervention to assess the sustainability of behavioral changes. Standardized questionnaires will be administered to adolescents and their parents or guardians to measure changes in knowledge, attitudes, and behaviors related to food literacy and adherence to MD. Data will also be gathered on demographic and socioeconomic characteristics, dietary intake, cooking and gardening practices, food preferences, sustainability perceptions, and family food routines to evaluate knowledge transference from school to home. In addition, objective outcome measures, such as anthropometric indicators (e.g., BMI), blood pressure, and, when feasible, dietary biomarkers, will complement self-reported data and enhance reliability. To further strengthen the mixed-methods approach, qualitative data will be collected through structured focus group discussions and in-depth interviews in the selected intervention schools [55,56]. The participants will include adolescents, parents, and teachers. The topics focus on perceived benefits and challenges, experiences with various components, and suggestions for improvement. Structured observations of cooking and gardening sessions, as well as 24 h dietary recalls, will be conducted to provide richer contextual and behavioral insights. Process evaluation will be conducted in parallel to assess the fidelity and quality of implementation across different school settings, using teacher checklists, student activity logs, and on-site observations by the project team. This will help to identify the most effective and feasible components of the intervention and inform future scalability. Moreover, any concurrent health or nutrition initiatives in participating schools or communities will be documented and considered in the analysis to minimize confounding effects. The influence of the home food environment will also be examined through additional modules that assess family dietary patterns and household support for healthy eating practices. All quantitative data will be analyzed using appropriate statistical models, including cluster analysis and multilevel modeling, to account for the nested structure of the data (students within schools) and control for school-level variation. Thematic analysis will be applied to qualitative data to identify emerging patterns and deepen the interpretation of the quantitative findings. This comprehensive evaluation strategy will provide robust evidence on the reach, effectiveness, sustainability, and perceived value of FOODWISELab: The Mediterranean Diet Experience, offering critical insights for its refinement, future scaling up, and integration into broader educational and health policies.

**Figure 2:**
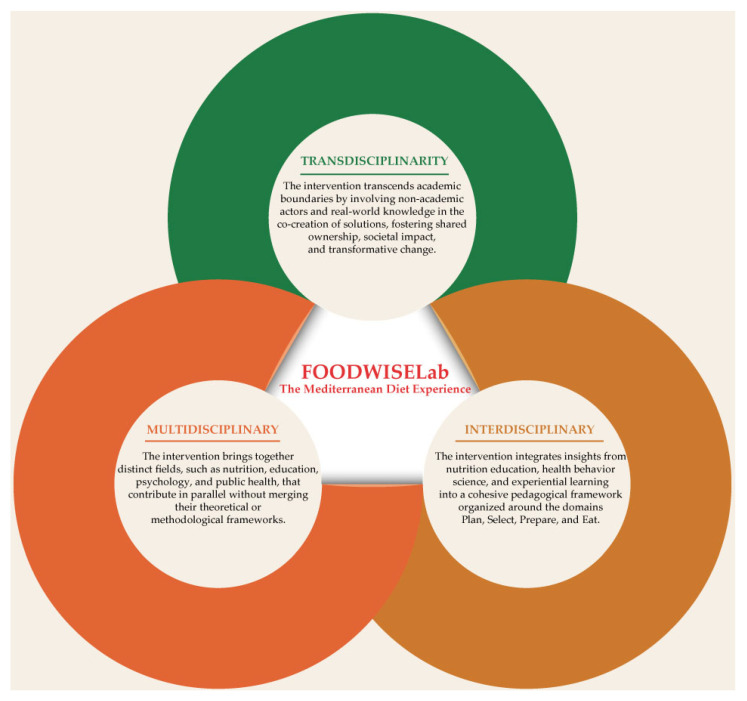
Knowledge Integration in FOODWISELab: The Mediterranean Diet Experience. This diagram illustrates how FOODWISELab is shaped by multidisciplinary, interdisciplinary, and transdisciplinary approaches—combining contributions from diverse academic fields, integrating knowledge into a cohesive pedagogical model, and engaging non-academic actors to co-create culturally meaningful food literacy solutions.

FOODWISELab: The Mediterranean Diet Experience serves as a paradigm of a comprehensive approach to food literacy education, integrating multidisciplinary, interdisciplinary, and transdisciplinary components (Figure 2). From a multidisciplinary standpoint, the initiative unites various fields, such as nutrition, education, psychology, public health, social sciences, environmental sciences, digital technology, and pedagogical design, each contributing distinct expertise while maintaining their disciplinary boundaries. Conversely, its interdisciplinary nature is demonstrated through the integration of concepts from nutrition education, health behavior science, and experiential learning within a unified pedagogical framework centered on four domains: planning, selecting, preparing, and eating. This is further supported by the incorporation of school gardens, cooking workshops, digital tools, and community engagement, alongside the implementation of a randomized controlled trial that combines epidemiological, educational, and behavioral evaluation methodologies. This project embraces a transdisciplinary approach by actively engaging students, families, teachers, and local communities in the co-creation of knowledge through workshops and collaborative activities. This approach facilitates intergenerational learning, reinforces cultural traditions, and extends the program’s impact beyond the classroom into everyday life. The digital platform acts as a conduit between academic content and real-world experiences, promoting continuous learning, and supporting cultural sustainability. Collectively, these three approaches highlight the project’s transformative potential and ability to inform educational policies and practices across diverse social and cultural contexts [57,58].

## 4. Conclusions

This paper underscores the urgent need to address the insufficient levels of food literacy among adolescents, particularly in Mediterranean countries such as Portugal, where adherence to traditional dietary patterns is declining, while the rates of overweight and obesity continue to rise. Although the existing literature highlights the efficacy of experiential school-based interventions, especially those rooted in local food cultures, few studies have translated this knowledge into structured context-sensitive programs. This review contributes to the field not only by synthesizing current evidence, but also by proposing a concrete and theoretically grounded intervention model: FOODWISELab: The Mediterranean Diet Experience. This protocol represents a strategic translation of research into practice and offers a culturally relevant and developmentally appropriate framework tailored to the Portuguese context. By organizing learning around four pedagogical domains— planning, selecting, preparing, and eating —and integrating school gardening, cooking workshops, digital tools, and family and community engagement, the intervention addressed both the cognitive and behavioral dimensions of food literacy. Importantly, this protocol fills the documented gap by offering a feasible and replicable model that can be adapted to diverse educational and cultural settings. Its potential is particularly strong in other Mediterranean countries, where shared food traditions and dietary patterns can support culturally grounded implementation. Its structured design and comprehensive scope, supported by a robust evaluation plan, make it a valuable tool for guiding future policies and educational practices. By embedding the principles of the MD within a participatory and meaningful learning experience, FOODWISELab: The Mediterranean Diet Experience promotes not only health and sustainability, but also the preservation of cultural heritage. Ultimately, this review goes beyond identifying a need: it offers an actionable solution with the potential for real-world impact, reinforcing the importance of locally meaningful food literacy initiatives that empower adolescents and support lifelong healthy behaviors.

## Figures and Tables

**Table 1:** Overview of school-based cooking interventions targeting adolescents (aged 12–18).

Study	Intervention Description	Duration	Outcomes
Beets et al. (2007) [41]	‘Culinary Camp’ Summer Cooking Program with cooking demos and group dish preparation.	Eight days, four-hour sessions daily.	Improved knowledge (*p* = 0.03) and cooking ability (*p* = 0.04); reduced negative attitudes; no significant change in frequency (*p* = 0.34).
McAleese and Rankin (2007) [40]	Garden-based education: gardening, cooking, cookbook creation, vegetable-based meals.	Twelve-week period.	Greater fruit/veg intake, improved intake of vitamins A, C and fiber (*p* < 0.001).
Chessen et al. (2009) [37]	Pink and Dude Chefs program: knife skills, balanced meals, nutrition panels, portion sizes.	Six weeks; 30 min lecture +90 min practicum per session.	Outcomes not stated in the document.
Gatenby et al. (2011) [36]	After-school cooking clubs focusing on multicultural recipes.	Ten 1.5 h clubs over 10 weeks.	Improved meal prep skills, ability to cook healthy meals (*p* < 0.05), increased cultural awareness.
Evans et al. (2012) [39]	Sprouting Healthy Kids: school garden, nutrition sessions, taste testing, farm visits.	10 weeks; 3 × 1 h nutrition sessions +4 × 45 min garden/week.	Higher self-efficacy, knowledge, reduced preference for unhealthy foods, increased fruit/veg intake (*p* < 0.05).
Thomas and Irwin (2013) [42]	Cook It Up!: community-based cooking with chefs, focus on self-efficacy and food literacy.	15 months.	Key facilitators of success: aptitude, food literacy, local ingredients; barrier: access to fast food.
Sullivan et al. (2025) [38]	Food Literacy Boot Camp: nutrition education, cooking, food safety, physical activity knowledge.	Four afternoons (~12 h), split into two 5-day sessions.	Reduced sugary drinks, better eating out choices, improved PA, handwashing, food hygiene; qualitative improvements in knowledge, engagement, skills.
